# Effectiveness of Digital Health Interventions (DHI) in Chronic Pain Management: A Scoping Review of Current Evidence and Emerging Trends

**DOI:** 10.7759/cureus.72562

**Published:** 2024-10-28

**Authors:** Shannon Weatherly, Tara McKenna, Simon Wahba, Arielle Friedman, Wesley Goltry, Talha Wahid, Hussein Abourahma, Kenneth Lee, Ahmed Rehman, Ali Odeh, Joshua Costin

**Affiliations:** 1 Dr. Kiran C. Patel College of Osteopathic Medicine, Nova Southeastern University, Fort Lauderdale, USA; 2 Department of Medical Education, Dr. Kiran C. Patel College of Allopathic Medicine, Nova Southeastern University, Fort Lauderdale, USA

**Keywords:** digital health technology, digital therapeutics, mobile health technology, pain assessment, remote healthcare, wearable devices, wearable technology

## Abstract

This review aims to address the impact of digital health interventions (DHIs) on chronic pain management, specifically, mHealth, eHealth, wearable devices, virtual reality (VR), and artificial intelligence. The following study identifies and assesses DHIs’ efficacy in specific chronic pain conditions and then extrapolates improved outcomes and patient groups that benefit from their use. Using a systematic methodology, this review synthesizes findings that could improve knowledge for patients and practitioners in chronic pain management while also addressing gaps in understanding the impact of DHIs. Sub-questions guide the identification of gaps and recommendations assessing DHIs' effectiveness for pain reduction and improved quality of life.

A systematic search across databases (EMBASE, Ovid MEDLINE, CINAHL, Web of Sciences, Cochrane Central) targets original, English-language, peer-reviewed studies (2013-2023). The inclusion criteria cover DHIs in chronic pain management for adults age 18+, excluding non-full texts, reviews, opinion pieces, or unrelated articles. Search terms include “chronic pain management” OR “long-term pain relief strategies” OR “sustained pain alleviation” OR “pain control for chronic conditions” OR “chronic pain” AND “ehealth interventions” OR “mobile health interventions” OR “digital therapy” OR “health informatic solutions” OR “digital health intervention.” After applying inclusion criteria, 34 articles from 11 countries are included, with studies conducted primarily in the United States (n = 17), United Kingdom (n = 4), and Australia (n = 3).

DHIs are emerging as effective tools in pain management, as they can emphasize patient autonomy and communication with clinicians while enabling medical self-management in diverse populations. These various digital interventions show promise in reducing pharmaceutical usage and deferring surgical procedures, with most studies reporting positive outcomes in pain reduction. DHIs were also associated with positive mental health outcomes; however, some studies found no significant improvement. Additionally, interventions targeting pain catastrophizing showed varied results, with some app-based approaches demonstrating promise. Overall, the review underscores the potential of DHIs in improving chronic pain management outcomes.

## Introduction and background

Within the last decade, the development of medical technology has revolutionized the healthcare landscape at an exponential pace. From imaging studies to expedited billing processes, aspects of medicine that were once monotonous and burdensome are now achievable through a few clicks of a button. The rapid growth of technological advancements in healthcare and the widespread availability of innovative portable electronic devices introduce opportunities to transform the future for all patients [1]. 

Chronic pain is a condition estimated to afflict over 50 million Americans (roughly one in five adults) and 1.5 billion individuals worldwide [2]. Chronic pain is defined by the International Association for the Study of Pain (IASP) as “persistent or recurrent pain lasting longer than 3 months” [2]. Although pain constitutes only a symptom and is not a formal diagnosis, there are numerous conditions that are intimately entwined with chronic pain, including arthritis, fibromyalgia, migraines and headaches, inflammatory bowel disease (IBD), low back pain (LBP), neuropathy, chronic fatigue syndrome (CFS), endometriosis, and chronic postsurgical pain [3,4]. Arthritis refers to the inflammation and/or degeneration of joints, often caused by autoimmune processes or "wear and tear" [5]. Fibromyalgia is a chronic neurological disorder characterized by diffuse musculoskeletal pain with associated symptoms of fatigue, sleep disturbances, cognitive difficulties, and heightened sensitivity to pain [6]. Migraine is a debilitating type of headache characterized by “recurrent attacks of moderate to severe throbbing and pulsating pain on one side of the head” [7]. IBD is a functional gastrointestinal disorder characterized by recurrent abdominal pain or discomfort and changes in bowel habits such as diarrhea, constipation, or a combination of both [8]. LBP is defined as “a painful neurological disorder that affects the lower segment of the spine,” and it is one of the most common causes of physician office visits [9,10]. Neuropathies are caused by nerve damage or dysfunction of peripheral nerves, resulting in persistent pain that is often described as burning, tingling, or shooting [11]. CFS, also known as myalgic encephalomyelitis, is a multifaceted condition characterized by extreme fatigue with associated muscle pain, aches, joint pain, and headaches [12,13]. Endometriosis is a chronic gynecological disorder resulting from extra-uterine proliferation of uterine tissue, causing chronic pelvic pain and impaired fertility [14,15]. Chronic postsurgical pain is “pain persisting at least three months after surgery, that was not present before surgery, or that had different characteristics or increased intensity from preoperative pain, localized to the surgical site or a referred area" [16,17]. 

Although the underlying causes of these conditions vary, they all share a common thread: persistent pain. Normally, pain serves a crucial protective function, but in the context of certain diseases, chronic pain can significantly impact quality of life [18]. Chronic pain often arises from immune activation, inflammation, or irritation of nearby tissues, nerves, and organs. Persistent inflammation sensitizes nociceptive and non-nociceptive sensory nerve fibers, leading to a lowered pain threshold-a phenomenon known as peripheral sensitization [18]. Prolonged exposure to pain can also lead to the brain interpreting pain signals as more intense, a process called central sensitization [19,20]. Most postoperative pain falls under the category of "nociceptive pain," which stems from tissue damage [21]. Furthermore, chronic pain may have psychological effects such as depression and anxiety, which can heighten pain perception and contribute to a state of heightened sensitivity [19, 22]. 

Treatment and prevention of chronic pain presents a multifaceted challenge due to the complex nature of pain generation and chemical signaling in various disease states [23,24]. Management of chronic pain differs depending on the underlying condition and often encompasses a variety of approaches aimed at mitigating the impact of the pain on an individual’s quality of life. Physicians frequently rely on pharmacologic interventions to manage symptoms, such as over-the-counter nonsteroidal-anti-inflammatory drugs (NSAIDs) and prescription analgesics (including opioids) [25]. Adjunctive treatments include antidepressants, anticonvulsants, and surgical interventions in refractory cases [25,26]. 

A challenge with many chronic diseases and associated pain is that appropriate treatment methodologies may be limited by the patient’s memory of their symptoms, pain severity, and frequency of discomfort. Patients often struggle to accurately recall their pain levels and experiences, leading to less effective treatments. Physicians aim to alleviate these discrepancies through the use of self-reported pain scales and questionnaires, but such methods have limitations because they rely on the patient's subjective reporting [27]. The emergence of modern digital health interventions (DHIs) such as electronic health (eHealth) and mobile health (mHealth) aims to improve this process, which invites opportunity for promising advances in the future of chronic pain management. 

"eHealth" is a catch-all term referring to the various aspects of healthcare and related technologies that have been advanced through creation of the internet and communication technology [28]. DHIs and digital health are concepts that fall under the umbrella of eHealth. The World Health Organization defines "digital health" as “the use of information and communication technology in support of health and health-related fields” [24]. DHIs, a form of digital health, are “discrete functionalit[ies] of digital technology [used] to achieve health-related objectives”. DHIs include mobile applications, virtual reality (VR) programs, artificial intelligence, and wearable devices [29]. Wearable devices are a type of wearable health technology that provide real-time feedback by continuously collecting data regarding a person’s physical activity, heart rate, sleep patterns, stress indicators, and monitor chronic disease progression [29]. mHealth is another subset of eHealth defined as the “use of mobile wireless technologies for public health” [2,24]. The use of digital health in the form of telemedicine is well-established and widely utilized in the post-COVID era, particularly for mental health treatment and follow-up. Still, there remains a gap in the established use and success of eHealth and mHealth for chronic pain management [30,31]. Therefore, a comprehensive assessment of the existing knowledge gap is imperative to not only ascertain the potential advantages of emerging DHIs in chronic pain management but also to lay a foundation for future related research. 

The emergence of eHealth and mHealth offers potential to improve symptom record-keeping and expand accessibility to cost-effective and convenient solutions for patients who are vulnerable to shortcomings of the US healthcare system [24]. DHIs provide a solution to ensure health objectives are achieved for patients with limited coverage, delayed access to care, or those who are geographically underserved [24]. DHIs can also prevent reduced quality care through facilitation of more accurate data collection and increased productivity of patient’s scheduled office visits [24]. Notably, adopting DHIs also offers an opportunity to address the opioid epidemic by improving efficacy of arranged treatments and thus reducing the need for opioid prescriptions. There is a well-established relationship between chronic medical conditions and an overreliance on pharmacological methods of pain management. Despite this notion, the American College of Physicians recommends nonpharmacological modalities as a first-line therapy for pain management in conditions such as chronic low back pain (CLBP) [9]. Existing literature explores different modes of DHIs in the management of singular chronic pain conditions and their respective management rather than evaluated as a whole, further emphasizing the need for a comprehensive evaluation of these devices. 

Is there a role for DHIs in monitoring, managing, and optimizing outcomes for chronic pain conditions? Despite an extensive search, no published reviews have comprehensively covered these various medical conditions collectively in the context of therapeutic responses to DHIs, which includes mobile apps, VR, and artificial intelligence. Gaps in research emphasizes the urgency of conducting a scoping review to navigate these growing technologies for the improvement in health outcomes and the reduction of suffering in patients living with chronic pain. 

As the digital health landscape rapidly expands, it is imperative that healthcare evolves in tandem with emerging technologies to enhance patient care, particularly in the context of chronic pain management. Chronic pain is often associated with debilitating consequences, including self-defeat, depression, and overall lower quality of life [32]. The primary objective of this scoping review is to examine peer-reviewed studies to investigate the recent advancements in DHIs for chronic pain management. The review seeks to identify conditions that exhibit improved treatment outcomes through the utilization of DHIs, patient groups most likely to benefit from DHIs, and the specific types of DHIs that have the potential to enhance the quality of life for chronic pain patients. 

## Review

Methods 

Objective 

This scoping review methodology was selected to facilitate a thorough synthesis and mapping of current evidence and emergent trends in DHI, such as eHealth and mHealth, to enhance pain management and contribute to shaping the future state of chronic pain research. This scoping review was performed consistent with guidance provided by the Joanna Briggs Institute Review Manual [33] and followed the Preferred Reporting Items for Systematic Reviews and Meta-Analyses Extension for Scoping Reviews (PRISMA-ScR) guidelines [34]. 

Eligibility Criteria 

The PCC model was used in which P stands for population, C stands for concept, and C stands for context. The population involved studies on individuals of any age 18+ and any gender, who are diagnosed with a condition associated with chronic pain. The concept focused on the use of DHI for effectively managing conditions frequently linked with chronic pain, as detailed in the background. Studies focused on chronic pain management beyond the specific disorders within the study’s scope were omitted from consideration. DHIs encompass a wide range of technologies and interventions that are delivered through digital platforms. The study focused on mobile apps, web-based tools, and wearable devices designed to manage chronic pain. Chronic pain management pertains to the various strategies, approaches, and interventions aimed at mitigating the impact of chronic pain on individuals' lives. For further identification of the study’s concept, effectiveness was related to the assessment of how well DHIs contributed to managing chronic pain. Effectiveness was identified to encompass various outcomes such as pain reduction, improved quality of life, enhanced functionality, patient satisfaction, improved patient and physician communication, and more. Additionally, current evidence refers to the existing body of research and literature that explored the effects of DHIs on chronic pain management, which included studies that have investigated the outcomes, benefits, and limitations of such interventions. Lastly, emerging trends within the study’s concept involved identifying and discussing new or evolving patterns, technologies, approaches, or strategies within our scope of DHIs for chronic pain management. The context of this review included chronic pain-associated conditions such as musculoskeletal conditions (arthritis, osteoarthritis, LBP), fibromyalgia, migraines and headaches, endometriosis, IBD, and CFS. Additionally, the types of DHIs will specifically relate to mHealth and eHealth, such as wearable devices, VR, mobile apps, and artificial intelligence. 

Information Sources and Search Strategy 

This scoping review included experimental and quasi-experimental study designs, comprising randomized controlled trials, nonrandomized controlled trials, and studies conducted before and after interventions. Additionally, analytical observational studies, such as prospective and retrospective cohort studies, case-control studies, and analytical cross-sectional studies, were incorporated. The review also considered the inclusion of descriptive observational study designs, such as case series, individual case reports, and descriptive cross-sectional studies. 

This scoping review utilized EMBASE, Ovid MEDLINE, Cumulated Index in Nursing and Allied Health Literature (CINAHL), Web of Science, and Cochrane Central. The key search terms included “chronic pain manage” OR “long-term pain relief” OR “sustained pain alleviation” OR “pain control for chronic conditions” OR “chronic pain” AND “ehealth interventions” OR “mobile health interventions” OR “digital therapy” OR “health informatic solutions” OR “digital health intervention.” Databases were accessed on October 6, 2023.

Studies were included in this review if they were original, published works focusing on chronic pain patients with one of the named conditions using DHIs as an adjunct of management. Additionally, studies that focused on trends of DHIs in pain management were included. Studies outside the English language, systemic reviews, books, or opinion pieces were excluded. Additionally, studies that did not primarily address the management of chronic pain conditions or did not investigate DHIs in patients with chronic pain will be excluded. Only studies published between 2013 and 2023, provided they are available in full text and have been formally published, will be considered for inclusion. 

Data Analysis 

For managing records and data throughout the review, JBI Sumari (https://sumari.jbi.global/) and MS Excel (Microsoft Corporation, Redmond, Washington, United States) software programs were utilized. Data was extracted from papers included in the scoping review by two or more independent reviewers. Extracted data included participant information, the core concept, context, study methods, and any other pertinent key findings. Data extraction was facilitated using an Excel spreadsheet. After conducting the data analysis, a PRISMA flow chart was utilized to present the steps followed in the search strategy. 

Quality Assessment 

The 34 articles selected from the full-text reviews were evaluated for quality using the JBI critical appraisal tools. Each article was evaluated by each author, and discrepancies between the scoring of the evaluators were discussed and resolved, with three authors ultimately agreeing upon a checklist score. Studies were included if they had a low risk of bias or better, defined as a score of 70% or above following JBI critical appraisal method. All 34 were included and had a low risk of bias according to the JBI checklists. 

Results 

**Figure 1:**
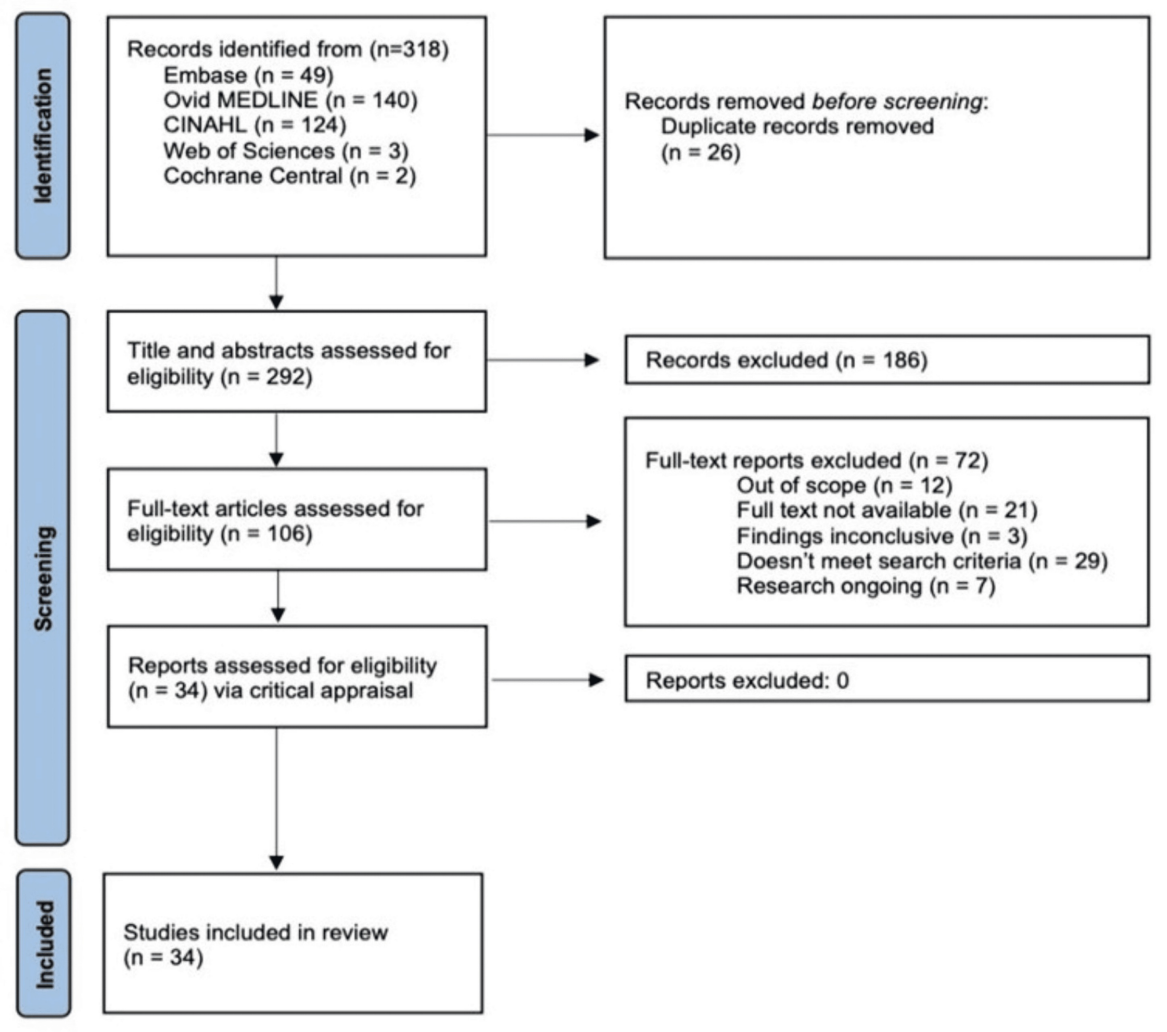
The Preferred Reporting Items for Systematic Reviews and Meta-analyses (PRISMA) flow chart The PRISMA flow chart tracks the article selection process leading to the inclusion of 34 articles in this review

After searching the databases using the predetermined search terms and applying the inclusion and exclusion criteria, 34 articles were identified as being within the scope of this review (Figure 1). All studies included in the review are summarized in Table 1. 

**Table 1:** Data extraction chart organized by condition CLBP: Chronic low back pain; DTxP: digital therapeutics for pain; ITT: intention-to-treat; FM: fibromyalgia; FM-ST: fibromyalgia-symptom tracking; FM-ACT: fibromyalgia-acceptance and commitment therapy; REN: remote electrical neuromodulation; GIER: guided intervention of education and relaxation; TKR: total knee replacement; MCID: minimal clinically important difference; mAPA: mobile auricular point acupressure; PEG tool: pain, enjoyment, and general tool activity

Data extraction chart organized by condition		
Musculoskeletal pain (arthritis)		
Reference	Study design	Study findings
Kelly et al. [35]	Qualitative research	The study highlighted the benefits of a flexible blended care model combining in-person and eHealth services, with eHealth aiding in self-management and patient education but facing challenges in diagnosis and relationship-building. It emphasizes the need for personalized guidance to address concerns about attrition and adherence, recommending further testing of remote monitoring and behavioral change interventions
Bhattarai et al. [36]	Qualitative study using semi-structured interviews	Out of 18 invited participants, 16 agreed to participate in qualitative interviews, with a mean age of 73.2 years, mostly female, and primarily based in New South Wales. Four key themes emerged: apps are valuable but can be harmful, they must align with users' needs, clinician involvement is crucial, and the design should prioritize the end-user experience
Musculoskeletal (back pain)		
Reference	Study design	Study findings
Almhdawi et al. [37]	Double-blind randomized controlled trial	After six weeks of using the "Relieve my back" app, the experimental group showed significant reductions in pain and disability, along with improved quality of life compared to the control group. The app appears to be an innovative and effective method for treating non-specific low back pain in office workers, though further research with larger samples and longer follow-ups is recommended
Amorim et al. [38]	Randomized controlled trial	Out of 90 invited participants, 68 joined the study, with a high response rate of 89% for weekly questionnaires and a satisfaction rating of 8.7 out of 10. While the intervention group showed a 38% reduction in care-seeking compared to standard care, the difference was not statistically significant, and no differences were found in pain levels or activity limitation; the approach is feasible and well accepted, but further research is needed
Chhabra et al. [39]	Randomized controlled trial	Chronic low back pain (CLBP) is a common issue with significant disability and economic impact, and while exercise is effective, adherence remains a challenge. This study evaluated the Snapcare App, designed to improve exercise adherence in CLBP patients, finding that while both the app and conventional groups experienced significant reductions in pain over 12 weeks, the App group showed a significantly greater decrease in disability, highlighting its potential to enhance patient outcomes
Eccleston et al. [40]	Prospective double blind, randomized controlled trial	Out of 72 participants assessed for eligibility, 34 were randomized into three groups: 11 to digital therapeutics for pain (DTxP), 12 to placebo sham, and 11 to standard care. After a second randomization, 8 standard care participants were reassigned to either the DTxP or sham placebo group, resulting in 42 participants (5 male, 37 female, average age 54.69 years) included in the intention-to-treat (ITT) analysis, all of whom were white, with one identifying as Hispanic or Latino; participants had an average baseline pain intensity of 5.56, with most reporting back pain lasting over 5 years
Irvine et al. [41]	Randomized controlled trial	The study evaluated the “FitBack” mobile-web intervention for managing and preventing lower back pain, finding that users experienced significantly better outcomes in pain levels, functionality, quality of life, and well-being compared to both the control and alternative care groups at 4-month follow-up. FitBack users showed greater improvements in physical, behavioral, and worksite measures and were less likely to report current back pain, demonstrating the potential of this self-tailored, theoretically based intervention as a cost-effective tool for large-scale self-management of low back pain, although further research is needed to assess long-term use and engagement
Madill et al. [42]	Cohort study	In a beta test involving 30 older adults with chronic low back pain, the app was well-received for its ease of use and reliability. Participants appreciated its usefulness in understanding their condition and communicating with physicians, though some found the information redundant. The positive feedback suggests the app has potential for supporting older adults with CLBP, though additional research in larger and more diverse groups is needed
Ross et al. [43]	Retrospective study	In a retrospective analysis of 461 participants using the selfBACK app for chronic pain management, those with higher engagement reported more pain, activity interference, and disability, while those managing pain better engaged less with the app. The study indicates that while the app was easy to use, higher engagement might not always correlate with better outcomes, suggesting that future apps should be more adaptable and tailored to individual needs to improve adherence and effectiveness in chronic pain management
Rughani et al. [44]	Randomized controlled trial*	The study found that individuals with higher depressive or stress symptoms reported more severe baseline low back pain (LBP)-related disability and lower self-efficacy, but LBP-related disability improved over time regardless of these symptoms. The selfBACK app was effective in enhancing outcomes for all users, including those with high depressive or stress symptoms, though initial satisfaction and engagement were lower for these individuals. Overall, the selfBACK app appears beneficial for managing LBP, even among patients with elevated depressive or stress symptoms
Sandal et al. [45]	Randomized controlled trial	In a study with 461 lower back pain (LBP) participants, those using the SELFBACK app showed a modest improvement in Roland–Morris Disability Questionnaire (RMDQ) scores after 3 months compared to the control group receiving usual care, with an adjusted mean difference of 0.79 favoring the app. While 52% of the intervention group reported significant improvements versus 39% in the control, the clinical significance of these findings remains uncertain
Trujillo et al. [46]	Case series	Individual sessions of virtual embodiment training significantly reduced pain intensity in both patients and led to improvements in pain catastrophizing subscales. These findings suggest that virtual embodiment training can effectively alleviate chronic pain symptoms and enhance mobility and function, potentially through recontextualizing sensory feedback during virtual rehabilitation exercises
Vad et al. [47]	Prospective pilot study; cohort study	In a study of 75 patients with CLBP, the Back Rx app led to significant improvements, with pain scores decreasing from 5.17 to 3.8 on the VAS and enhanced functionality. Although app compliance was 52% and 65% of patients rated their experience positively, the results suggest that the app effectively reduced pain and improved functionality, highlighting the need for strategies to boost patient adherence
Pelvic pain (endometriosis)		
Reference	Study design	Study findings
Merlot et al. [48]	Randomized controlled trial	The study with 102 endometriosis patients compared the Endocare group to a sham control, finding that Endocare significantly reduced pain intensity more than the sham group on multiple days and time points. Endocare led to a maximum pain reduction of 51.58% by day 2, while the sham group had a 27.37% reduction by day 3. Both groups saw decreased medication use, and Endocare was effective and safe for self-administered at-home use, with no reported adverse events
Merlot et al. [49]	Self-administered RCT	Endocare, a virtual reality immersive treatment, significantly reduced pain intensity in women with endometriosis compared to a digital control, showing a mean pain decrease from 6.0 to 4.5 versus 5.7 to 5.0 in the control group. Endocare was more effective at reducing pain perception up to 4 hours post-treatment and provided higher perceived pain relief (28% vs. 15%). This pilot study suggests Endocare as a promising alternative to hormonal treatments or surgery, with further investigation planned for its repeated use in a larger population
Fibromyalgia		
Reference	Study design	Study findings
Catella et al. [50]	Randomized active-controlled study	The study screened 106 patients with primary fibromyalgia (FM), randomizing 67 participants to either FM-ACT or FM-ST treatment groups. The study population, predominantly female with an average age of 53, was well-matched demographically and clinically between groups, though the fibromyalgia-Symptom Tracking (FM-ST) group had a longer duration since diagnosis, and the fibromyalgia-Acceptance and Commitment Therapy (FM-ACT) group had more participants using gabapentin; study attrition was minimal, with only a few withdrawals
Migraine & headache		
Reference	Study design	Study findings
Buse et al. [51]	In a two-arm observational study- cohort study	Remote electrical neuromodulation (REN) is an FDA-cleared nonpharmacological migraine treatment. This study found that combining REN with a tailored behavioral therapy, guided intervention of education and relaxation (GIER), enhances therapeutic efficacy, leading to greater reductions in pain and disability compared to REN alone
Other musculoskeletal (non-back-related musculoskeletal pain)		
Reference	Study design	Study findings
Bedson et al. [52]	Feasibility study	In a study on newly developed app, well-received by both patients and general practitioners, provided a novel way to discuss pain control, with users finding it easy to use and interpret. The app showed strong correlations with validated pain questionnaires, indicating its accuracy in monitoring musculoskeletal conditions in response to analgesic prescribing; future work should explore how tracking pain trajectories can support self-management
Janela et al. [53]	Cohort study	The study involved 336 participants, with 296 starting the program and 79.1% completing it. Baseline characteristics were similar between completers and non-completers, except for age, with completers being older. Latent Growth Curve analysis showed significant improvements in QuickDASH scores (51.6% recovery) and pain reduction (54.8% improvement). Participants who used the app more frequently had better outcomes. Medication usage decreased, surgery intent reduced by 55.5%, and mental health improved. Engagement was high, with participants performing 2.7 sessions per week and high engagement associated with greater improvements in QuickDASH and pain. Overall, the program was effective in improving disability, pain, and productivity, while also reducing the burden of chronic shoulder conditions
Mecklenburg et al. [54]	Randomized control Trial	The study involved 30 older adults with CLBP, who found the app highly usable and engaging. Most participants appreciated its ease of use and reliability, noting that it provided valuable new information and improved communication with their physicians, despite some overlap with existing knowledge. The app’s positive reception suggests it could effectively support older adults with CLBP, though further research in larger and more diverse populations is needed to confirm its broader efficacy
Pach et al. [55]	App-based randomized controlled trial	The study enrolled 220 participants with a mean age of 38.9 years and baseline neck pain of 5.7 points. Over 3 months, both the intervention and control groups experienced a reduction in neck pain, but no significant differences were observed between the groups. By week 12, only 40% of participants in the intervention group continued using the app for exercises
Pak et al. [56]	Randomized controlled trial	In a study comparing digital and conventional physical therapy for 90 participants, both groups showed significant functional improvements with no differences between them. Although the conventional therapy group experienced slightly greater reductions in average and least pain, the small effect sizes suggest these differences are not clinically meaningful. Both therapies had high adherence and satisfaction rates, and no adverse events were reported, indicating that remote digital programs can be an effective alternative to traditional in-person rehabilitation
Pronk et al. [57]	Unblinded RCT study	In a study evaluating the PainCoach app versus a control group post-total knee replacement (TKR), no significant differences in pain scores were observed between the groups. However, PainCoach users reported a 23.2% reduction in opiate use and a 14.6% increase in acetaminophen use, with active app engagement leading to faster pain relief and reduced use of opiates and gabapentin. Despite these findings, limitations such as an underpowered analysis and lack of cost-effectiveness assessment suggest the need for further research with larger, more diverse populations
Scheer et al. [58]	Longitudinal cohort study	In a study with 9,992 participants from urban and rural areas across the U.S., a 73.8% completion rate was achieved, with both groups showing high satisfaction and similar engagement with exercise sessions. Rural residents had higher engagement with educational content and better program completion rates, but both urban and rural groups experienced significant improvements in pain, mental health, and work productivity, with similar percentages meeting the minimal clinically important difference (MCID)
Yeh et al. [59]	Randomized controlled trial	The study evaluated the feasibility and effectiveness of an auricular point acupressure smartphone app, mobile auricular point acupressure (mAPA), for managing chronic musculoskeletal pain, comparing self-guided mAPA, in-person mAPA, and a control group. Both mAPA groups showed significant improvements in physical function and pain intensity, with the self-guided mAPA group achieving higher gains. Over 50% of participants in each mAPA group experienced at least a 30% reduction in pain, and around 80% were satisfied with the treatment, indicating that the mAPA app is a valuable and effective tool for pain self-management
Chronic pain (unspecified)		
Reference	Study design	Study findings
Bhatia et al. [60]	Multi-site trial; cohort trial	Of the participants, 73.6% agreed to use the app, with 63.4% of them continuing for at least one month. Those who used the app reported lower anxiety and a greater reduction in pain catastrophizing compared to those who did not, indicating that the MMP app may benefit patients with chronic pain, though further research is needed to explore factors influencing sustained engagement and outcomes
Guillory et al. [61]	Randomized controlled trial	The social support intervention delivered via SMS text messages significantly reduced pain perceptions, pain interference, and improved positive affect in chronic non-cancer pain patients compared to controls, with married or partnered patients benefiting the most. This pilot study demonstrates the feasibility and potential of mobile apps in pain management, highlighting their accessibility and effectiveness in reducing daily pain experiences through social support
Hogan et al. [62]	Quantitative study	Out of 1507 eligible veterans, 393 (26.1%) completed the baseline survey, with 236 (60.1%) finishing follow-up surveys and 20 veterans interviewed. Among survey respondents, 10.2% used the Pain Coach app, which was reported as easy to use by 58% of them, though usability issues were noted; app users showed greater pain self-efficacy and lower pain interference compared to non-users. The main barrier to app use was lack of discussion with healthcare teams, suggesting that future efforts should focus on increasing healthcare team endorsement to boost adoption and further study the app's impact
Ireland et al. [63]	Prospective pilot study (quantitative study)	The study achieved excellent compliance with an 80% retention rate and minimal need for repeating monitoring periods. Users consistently reported pain data and provided diary entries, with significant improvements observed in pacing activities, reduced overactivity, stabilized pain levels, and increased productivity; notable outcomes included a 20% average reduction in opioid use and positive consumer feedback, with all participants recommending the Pain ROADMAP program
Jamison et al. [64]	Randomized controlled trial	The study evaluated the impact of a smartphone pain app that allows chronic pain patients to assess, monitor, and communicate their status to providers. While patients with 2-way messaging used the app more and submitted more daily assessments, the differences were not significant; overall, 85.7% of participants believed the app improved practice, and 71.4% were satisfied with the app's summary graphs and its role in helping patients understand their pain
Jamison et al. [65]	Qualitative research	The study included 144 participants with an average age of 52 years, predominantly female, with various pain sites and an average BMI of 29.6. Participants used the pain app for an average of 84.1 daily entries, and their daily assessment scores showed significant correlations with perceived change in their condition. After 40 days, participants were classified based on 5 daily assessments: 9% as worse, 9% as better, and 82% as the same. Those classified as worse reported higher pain intensity, activity interference, and irritability, and had significantly higher pain catastrophizing, disability, and mood disturbance scores compared to other groups
Suso-Ribera et al. [66]	Randomized controlled trial	The study demonstrates the effectiveness of mobile apps, like the Pain Monitor, in providing precise daily assessments of chronic pain, offering a more accurate picture of treatment response compared to traditional retrospective methods. It highlights the potential of telemonitoring to impact physical and mental health outcomes in chronic pain patients and suggests that integrating alarm systems for immediate responses may enhance the effectiveness of telemonitoring. Additionally, the study provides valuable insights into managing physician burden and technology challenges, which could benefit future research, clinical practice, and policymaking in mobile health
Suso-Ribera et al. [67]	Validation/feasibility study	The Pain Monitor app was developed based on IMMPACT guidelines and existing reviews to assess its validity, reliability, feasibility, and usefulness for chronic pain management. It utilized ecological momentary assessment to capture a range of factors, including pain intensity, mood, and medication use, through repeated app-based assessments, traditional measures, and phone calls. This approach allowed for a thorough evaluation of the app’s performance and practicality in real-world settings
Zhao et al. [68]	Qualitative research study	The study reviewed 36 pain management apps, most of which functioned as pain diaries recording pain intensity, location, and impact on daily life. Despite their functionality, these apps had usability issues, including minimal involvement of healthcare professionals in their development, lack of cross-platform support, and non-compliance with standards like HIPAA. Additionally, none provided clinicians with graphical pain data visualization or supported the Pain, Enjoyment, and General Activity (PEG) tool for chronic pain management

**Figure 2:**
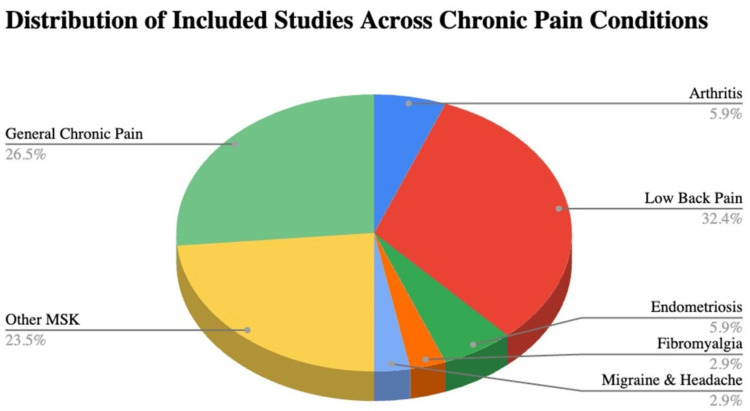
Distribution of included study relative to chronic pain condition type

From the data extraction list, a breakdown of chronic pain condition type by study was created. The studies included encompassed a variety of chronic pain disorders as shown in Table 1 with a breakdown of included studies by type of chronic pain shown in Table 2 and Figure 2. The largest proportion of articles that met the inclusion criteria were for musculoskeletal pain. Musculoskeletal pain was further categorized into two categories: back pain and non-back-related musculoskeletal pain. Ultimately, no studies on CFS and neuropathy were identified for inclusion. 

**Table 2:** Breakdown of articles included for each type of chronic pain

Type of chronic pain	# of included studies
Arthritis	n = 2
Back pain	n = 11
Chronic fatigue syndrome	n = 0
Endometriosis	n = 2
Fibromyalgia	n = 1
Inflammatory bowel disease	n = 0
Migraine & headache	n = 1
Neuropathy	n = 0
Other musculoskeletal (non-back-related musculoskeletal pain)	n = 8
Unspecified	n = 9
TOTAL	n = 34

Among 34 studies, 24 were quantitative [37-41,44,45,47-50,54-64,66], four were qualitative [36,51,35,68], and six used mixed methods [42,43,46,52,53,67]. Most studies included used surveys for collecting data [37-39,41,43,44,49,52-54,57-60,62,64]. The questionnaires used in surveys were mostly developed by Short Form Health Survey, University of Sydney; Keele University’s Institute for Primary Care and Health Sciences; Patient Health Questionnaire; Roland-Morris Disability Questionnaire; Survey of Pain Attitudes; Coping Strategies Questionnaire; Quick Disabilities of the Arm, Shoulder, and Hand Questionnaire; Knee Injury and Osteoarthritis Outcome Score Pain subscale; Endometriosis Health Profile; Wisconsin Brief Pain Questionnaire; and Pain Self-Efficacy Questionnaire. In the mixed-method studies, most commonly, content or thematic analyses were used. A total of two studies conducted in-depth or semi-structured interviews [36,62]. All 34 studies included had clear ethical statements within the paper either stating ethical board approval or exemption [35-68]. 

Characteristics of the Reviewed Studies 

**Figure 3:**
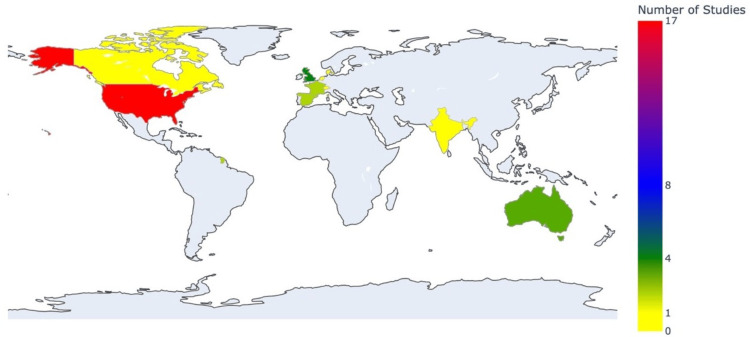
Distribution of studies analyzed by country origin

The studies were conducted in 11 countries, the majority being based in the United States (n = 7), the United Kingdom (n = 4), and Australia (n = 3). Other countries with less than three studies were Canada (n = 1), Denmark (n = 1), France (n = 2), India (n = 1), Jordan (n = 1), Netherlands (n = 1), Spain (n = 2), and Switzerland (n = 1) (Figure 3). 

Participants in the reviewed studies were from diverse subsets of conditions associated with chronic pain, which included arthritis (n = 2), fibromyalgia (n = 1), back pain (n = 11), other non-back-related musculoskeletal pain (n = 8), migraines and headaches (n = 1), endometriosis (n = 2), and unspecified chronic pain (n = 9) (Table 2, Figure 2). Significant gender differences were identified in three studies where the participants studied were primarily females [51,61,43]. 

Accessibility of DHIs 

Subtopics within the realm of DHI accessibility include enhanced patient autonomy and improved patient management of chronic pain conditions [44,50,54,60,63]. Positive pain outcomes were associated with the accessibility of DHIs, suggesting that improved adherence and frequent app usage are linked to better outcomes [68]. While DHIs simplify pain management and make it more accessible, two studies emphasize that these interventions should complement traditional therapies rather than replace standard care [38,68]. 

DHIs have also proven to enhance clinician accessibility, enabling providers to better manage patient pain [36,60,66,67]. Apps have demonstrated capabilities in tracking alarm symptoms (such as chest pain, vomiting, numbness) [65,66] and facilitation of two-way communication between doctors and patients [64]-features positively received by patients. The utilization of apps for tracking alarm symptoms has been associated with reduced anxiety for both clinicians and patients [66,67]. The underutilization of DHIs by providers was also found to have a negative impact on patient experiences due to unmet expectations of provider responsiveness, emphasizing the importance of clinician involvement in DHI design [36,64,68]. 

Furthermore, DHIs have expanded the accessibility of self-management tools to a broader spectrum of patients [41,59]. Particularly in regions with limited access to healthcare facilities, such as rural areas, DHIs offer easily accessible self-management tools that prove beneficial for patient outcomes [58]. The studies suggested that there was a decrease in care-seeking activities in groups that used DHI s [38]. The overarching goal of DHIs is to reduce the number of clinic and emergency room visits, saving patients from unnecessary trips and alleviating the burden on the healthcare system [65]. 

Reduced Healthcare Costs 

Reductions in healthcare costs with DHI use include overall cost reductions, reductions in pharmaceutical-associated costs, and savings resulting from the deferral of invasive procedures. Six studies reported a reduction in healthcare costs associated with the implementation of a DHI in chronic pain management [41,47,54,56,57,60,63]. Among them, two studies highlighted cost reductions by applications designed to manage different types of LBP [47,56]. The remaining 26 studies either lacked substantial evidence supporting cost reduction or did not use cost reduction/effectiveness as a metric for gauging the success of the management interventions. Notably, no studies reported an increase in associated healthcare costs. 

Multiple studies evaluated the decrease in healthcare costs achieved through a reduction in pharmaceutical usage, particularly opiate use [57,60,63], illustrating overlap between reduction in healthcare costs and accessibility associated with DHI use. The observation of diminished pharmaceutical use suggests potential cost savings linked to the alleviation of dependency [57]. Another dimension of cost reduction involved the ability of DHIs to defer the need for surgical procedures, specifically over a course of multiple years [54]. However, challenges in patient adherence underscored the necessity for further adjustments and enhancements needed for DHIs to optimize patient usage and subsequent benefit. 

A distinct avenue of interest within the realm of cost-effectiveness involved the standardization of strategies disseminated through a large platform, such as a mobile app [47,56]. This approach aimed to educate a broad audience in techniques like self-relaxation, offering convenient at-home interventions, potentially obviating the need for in-person doctor visits, and empowering individuals to manage their health proactively, ultimately alleviating unnecessary short-term and long-term healthcare costs [47,56]. Similarly, the utilization of a mobile-web intervention to reach a diverse population showcased the potential for cost-effectiveness and positive user engagement in a wider array of different socioeconomic demographics [41]. 

Reduction in Pain 

Twenty-one studies reported positive outcomes in pain reduction [37,39,41,45-51,53,56-59,61,62]. These studies employed diverse interventions, utilizing the DHIs both as primary care and adjuncts, reflecting the complex landscape of chronic pain management. Interventions looking at pain intensity demonstrated statistically significant improvements in pain intensity, disability index scores, and overall quality of life [37]. These findings underline the potential benefits of personalized approaches to chronic pain management. While over 60% of the articles reported pain reduction, no articles reported an increase with associated pain management techniques. 

The effectiveness of mHealth interventions, such as applications promoting adherence to physical activity and self-management, showcased positive outcomes in chronic LBP patients [39]. Several interventions across 17 studies, such as GIER with REN treatments and ACT, reported multifaceted improvements in pain relief, functionality, and return to normalcy [37,39,41,45-51,53,56-59,61,62]. These technology-based interventions, including applications and virtual training, demonstrated positive impacts on pain outcomes. 

Mental Health Implications 

Eleven studies included assessed aspects of mental health in relation to chronic pain [37,40,41,43,53,56,58,60,61,65-67]. Utilizing DHIs was associated with positive mental health outcomes including significantly reduced depression and anxiety [58,60,66,67], reduced pain-associated fear avoidance [40,53], and improved affect [61], as well as improved emotional understanding of and overall attitude toward pain [41]. One study found improvement in lower back pain (LBP) outcomes after utilizing an AI-based smartphone app, even in participants with high baseline levels of depression and stress [44]. 

DHIs were also associated with significantly reduced daily opioid use and even complete opioid cessation [60,63,57]. Several studies found no positive mental health outcomes after utilizing DHIs [37,43,56,64]. Some DHIs encompassed additional behavioral features such as relaxation techniques, autogenic training, meditation, guided imagery [55], mood recording features [4], and adjunctive behavioral techniques such as cognitive behavioral therapy and guided relaxation [51,56], in addition to daily questionnaires related to anxiety, depression, and pain catastrophizing [60]. No studies reported negative impacts on mental health. 

Pain Catastrophizing 

Eight studies investigated the impact of digital technologies on pain catastrophizing in patients with chronic pain. All assessments of pain catastrophizing were measured using the pain catastrophizing scale (PCS) [43,46,49,59,60,64,65,66]. Among these studies, two focused on VR-based DHIs [46,49], while six investigated DHIs delivered through smartphone apps [43,59,60,64-66]. VR-based DHIs did not yield significant reductions in PCS scores among patients with LBP [46] or endometriosis [49]. However, one of these devices demonstrated that patients with higher PCS scores experienced greater pain intensity 60 minutes after undergoing their first VR treatment [49]. In contrast, app-based devices produced more varied results. Three studies reported significant results in PCS scores for those living with chronic musculoskeletal pain conditions [59,60,65]: two of them demonstrating significant reductions in PCS scores [59,60], whereas the remaining study demonstrated that scores increased with a greater number of assessments completed by participants [65]. Additional app-based DHI studies found no statistically significant reductions in PCS scores for chronic pain [43,64]. Notably, one specific app-based DHI exhibited high levels of construct validity in assessing PCS, serving as a valuable guide for managing interventions in chronic pain treatment [66]. 

Discussion 

The evidence presented in this review underscores the efficacy of DHIs in improving chronic pain management. In the analysis of themes, including pain reduction, accessibility, and cost-effectiveness, DHIs demonstrate promise for enhancing patient outcomes and accessibility to care, particularly for disadvantaged and underserved patient populations. DHIs offer the potential for reducing healthcare expenditures and addressing mental health dimensions such as anxiety, depression, and pain catastrophizing. 

Reduction in Pain 

DHIs provide a diverse array of interventions tailored to address chronic pain based on individual patient needs and preferences. The reduction in pain achieved through personalized pain management strategies, coupled with the increased accessibility of pain management services from the convenience of a patient's home, offers an avenue for integrating holistic strategies into clinical practices to effectively address chronic pain. 

Noteworthy advancements are observed in the realm of mHealth applications. These applications focus on encouraging patients to adhere to physical activity and self-management, showcasing significant efficacy, particularly in conditions such as chronic LBP [39]. By integrating patient accountability with convenient access to medically approved instructions, mobile applications likely enhance patient adherence, thereby further bolstering the pain reduction potential of DHIs. 

Guided intervention of education and relaxation (GIER) with remote electrical neuromodulation (REN) treatments and acceptance and commitment therapy (ACT), a form of cognitive behavioral therapy, have exhibited multifaceted enhancements in pain relief, functional capacity, and restoration of normalcy, underscoring the value of taking a holistic approach in the management of chronic pain [50,51]. Virtual training programs and VR have shown positive impacts on pain outcomes, suggesting a complementary role of technological interventions alongside traditional pain management strategies [46, 62]. While digital interventions have shown positive impacts for some patients, it’s notable to mention the lack of accessibility to these programs based on cost and access to the applicable technology for patients worldwide. Further research could be conducted to evaluate VR as an adjunct to standard care to weigh the efficacy of these programs with the imposed financial burden to patients. 

While these findings provide valuable insights, several gaps in knowledge warrant further attention. Firstly, there remains a need for further exploration into the mechanisms underlying the effectiveness of these DHIs. Additionally, the review highlights the need for more studies assessing the long-term efficacy and sustainability of such interventions as well as their scalability and applicability across diverse patient populations such as those in rural areas and low socioeconomic status. While mHealth interventions have shown promise, there is a lack of standardized protocols and guidelines for their development and implementation in chronic pain management. This gap underscores the importance of future research in establishing best practices and ensuring the quality and effectiveness of mHealth interventions. 

Accessibility 

Accessibility provided by DHIs is a valuable asset for individuals with chronic pain conditions. DHIs play a pivotal role in streamlining pain management, facilitating ease of access to healthcare for socioeconomically disadvantaged and rural regions [41,59]. This enhanced accessibility can be attributed to the large number of pain management mobile applications, readily available on numerous mobile app platforms that are increasingly offered as a complement to standard care. 

DHIs not only enhance patient accessibility but also provide healthcare providers with the means to monitor patients and access pertinent pain data [64]. This data, in turn, can inform care decisions during both in-office and telehealth visits, contributing to higher quality care and more informed decision-making by physicians, ultimately benefiting patient outcomes. The favorability of two-way communication within apps among patients mitigates anxiety associated with chronic conditions, fostering a sense of security through readily available access to healthcare professionals in case of emergencies. Alarm symptom tracking further contributes to anxiety reduction by guiding patients in making informed decisions about whether to seek emergency medical services, thereby reducing unnecessary healthcare facility visits and associated costs [38]. 

However, a noteworthy barrier to the wider adoption of DHIs lies in the underutilization of available technology by healthcare providers [36,64,68]. Patient reports indicate that a lack of engagement and app usage by physicians dissuades patient engagement, highlighting the importance of physician support to bolster the credibility of DHIs in public perception. Despite their proven effectiveness, DHIs require active endorsement and utilization by physicians to ensure patient adherence. The potential cost associated with monetized applications may still deter some patients, although numerous cost-free applications are currently available. It is also necessary to mention that accessibility to DHIs is largely limited to technology-literate individuals who own a smartphone, VR device, or other wearable device who frequently engage with the user interface to provide accurate health tracking data. Additionally, other DHI interventions, such as VR technology, remain financially prohibitive and are unlikely to be covered by insurance, restricting their usage and potential pain reduction benefits to higher income households. Further research could be conducted to evaluate VR as an adjunct to the standard care to weigh the efficacy of these programs with the imposed financial burden to patients. 

The escalating prevalence of smartphone ownership is evident in the United States, with 76% of individuals who earn an annual income below $30,000 possessing access to technology. Furthermore, this percentage rises to 87% for those earning between $30,000 and $100,000 annually, underscoring the potential for DHIs to reach a substantial portion of the US populace, surpassing the reach of traditional care methods alone [68]. Nonetheless, it is imperative to acknowledge existing barriers for individuals from lower socioeconomic strata. A total of 41% of individuals earning less than $30,000 annually report a lack of home broadband access, constraining internet utilization and impeding the full utilization of certain key features inherent to DHIs, such as two-way communication and real-time alarm symptom monitoring by healthcare professionals. Additionally, a quarter of the population in rural and lower-income areas lacks access to smartphones entirely, posing a potential obstacle to the seamless integration of these technologies, warranting attention as a public health concern. 

These findings reinforce the notion that while DHIs streamline pain management and offer a valuable tool for patients lacking access to traditional healthcare facilities, they should be regarded as complementary to, rather than a replacement for, traditional therapies [3,18]. Kelly et al. advocate for a blended care model that combines technology with face-to-face management to optimize monitoring and care, asserting that DHIs primarily contribute to enhancing self-management. 

Reduced Healthcare Costs 

The observed cost reduction associated with DHIs for managing LBP underscores their potential to significantly impact chronic condition care. These findings not only demonstrate the cost-saving capabilities of DHIs in LBP management, but also suggest broader implications for addressing other chronic conditions [47,56]. Understanding how DHIs contribute to cost reduction in LBP care can inform the development of tailored intervention strategies with guidelines for the management of additional chronic conditions, thereby improving patient outcomes and reducing healthcare expenditure on a wider scale. 

The reduction in pharmaceutical usage, particularly opiates, due to DHI use, is another significant aspect of cost reduction in chronic pain management [57,60,63]. By diminishing reliance on medications, DHIs offer a pathway to decreased healthcare expenditures. Furthermore, DHIs demonstrate the potential to defer the need for surgical interventions over extended periods, as indicated by longitudinal studies [54]. These findings hold promise for guiding future research aimed at understanding the broader impact of DHIs on patient outcomes and healthcare spending, suggesting potential avenues for large-scale cost savings and improved management of chronic pain conditions. 

Although not consistently emphasized in the literature, some studies reported promising results regarding healthcare cost reduction through the utilization of DHIs for chronic pain management [47,56]. This suggests that cost reduction may be an associated benefit even in studies where it was not explicitly addressed. 

These findings underscore a need for further research aimed at evaluating the degree of healthcare cost reductions associated with DHI use in chronic pain management. Given the prevalent economic barriers hindering healthcare accessibility for patients with chronic conditions, understanding the nuances of cost reduction associated with digital interventions is imperative for reducing the global economic impact of treating patients living with chronic pain. This endeavor holds the potential to refine digital healthcare interventions to better meet the needs of patients and healthcare systems alike, ultimately fostering improved accessibility and affordability of care for individuals with chronic conditions. 

The emphasis on cost reduction in DHIs highlights their potential benefits for patients, especially in managing chronic conditions. These findings underscore the capacity of DHIs to revolutionize chronic condition care by offering cost-effective alternatives to traditional treatment methods. 

Mental Health Implications 

The utilization of DHIs serves to bolster the efficacy of a multifaceted approach to managing chronic pain conditions, particularly by addressing mental health-associated symptoms. DHIs incorporate a comprehensive array of behavioral components, including relaxation modalities, autogenic training, meditation, guided imagery, mood tracking, CBT, and guided relaxation, among others. Crucially, DHIs integrate daily assessments aimed at targeting anxiety, depression, providing a holistic framework for addressing the complex interplay between mental health and chronic pain management [60]. By encompassing diverse components, DHIs offer a promising avenue for effectively managing chronic pain conditions while concurrently addressing mental health-related symptoms, thereby enhancing overall patient well-being and treatment outcomes. 

The adoption of DHIs presents a promising avenue for improving mental health outcomes within the management of chronic pain, as supported by a growing body of empirical research. Studies demonstrate significant reductions in comorbid depression and anxiety, as well as decreases in fear-avoidance behaviors associated with pain, improvements in affect, and enhanced emotional understanding and coping attitudes toward pain [40,41,53,58,60,61,66,67]. Notably, positive outcomes in managing LBP through AI-based smartphone applications, even among participants with elevated levels of depression and stress at baseline, underscore the potential of DHIs to address mental health concerns within chronic pain populations [44]. 

Moreover, DHIs have shown efficacy in reducing daily opioid consumption and facilitating opioid cessation [57,60,63], suggesting potential benefits in mitigating opioid dependency. However, it is important to note that some investigations have failed to observe significant improvements in mental health parameters following DHI implementation though none saw a deterioration in mental health [37,43,56,64]. 

These findings underscore the potential of DHIs as efficacious instruments for enhancing mental well-being among chronic pain sufferers. However, further research is warranted to elucidate the underlying mechanisms driving DHI-mediated effects on mental health outcomes and to identify optimal intervention strategies tailored to individual patient profiles. Additionally, future investigations should explore specific chronic pain syndromes, such as LBP, and vulnerable patient cohorts, particularly those facing socioeconomic barriers to healthcare access. By addressing these gaps, future studies can provide a comprehensive understanding of the landscape of DHIs in chronic pain management, ultimately informing evidence-based practices and promoting holistic well-being for patients. 

Pain Catastrophizing 

In this study, the utilization of DHIs for the management of pain catastrophizing demonstrated mixed results. Pain catastrophizing, characterized by distressing cognitive thoughts associated with pain conditions, is quantified based on helplessness, rumination, and magnification scores [43,46,49,59,60,64-66]. Although pain catastrophizing represents a significant method of quantifying the pain experience, only a limited number of studies evaluated this parameter with DHI use. One smartphone DHI study reported significant reductions in pain catastrophizing scores (PCS) among patients living with osteoarthritis and CLBP [46,60]. However, contrasting results emerged from other studies evaluating DHIs in musculoskeletal conditions, with some demonstrating nonsignificant reductions in chronic musculoskeletal PCS scores [59], no change in PCS scores in nonspecific chronic pain and LBP, respectively [43,64], or even significantly increased nonspecific chronic pain PCS scores [65]. The predominant result of these studies suggests that DHIs must be further evaluated to ascertain the true efficacy of DHIs in managing PCS, with a particular focus on their role in the management of musculoskeletal conditions. 

Notably, one study reported impressive levels of construct validity, which refers to the degree to which a test or instrument accurately measures the theoretical constructs related to the pain experience that it is intended to measure, in a smartphone-based DHI [66]. This suggests the potential for DHIs to enhance the identification of patients prone to pain catastrophizing and serve as a valuable tool in a provider’s approach to treatment for chronic pain patients [66]. Consequently, the use of DHIs for accurately identifying pain catastrophizing patients could revolutionize patient-physician interactions especially for individuals who experience heightened distress due to their condition. This presents an opportunity for a more comprehensive assessment and personalized treatment, fostering trust and rapport with patients, aligning with the core values of the osteopathic profession. 

VR-based DHIs represent a cutting-edge opportunity to address chronic pain patients. Given the interconnectedness of pain catastrophizing with the cognitive process of pain perception, advancements in the immersive VR technology experience offer an increasing opportunity to leverage these technologies to reshape how patients perceive pain [46]. Through physical engagement with therapeutic exercises and continuous visual feedback offered by VR technologies, patients can potentially restructure their understanding of pain, leading to reduced PCS scores [46]. Moreover, VR-delivered DHIs demonstrate a favorable safety profile [46,56]. However, the high cost associated with these innovative devices presents an obstacle to wider access and adoption of these devices. 

Despite the beneficial impacts listed above, further research is needed to understand the underlying mechanisms through which DHIs enhance patient outcomes and accessibility to care, reduce healthcare expenditures, and address mental health dimensions of care, such as anxiety, depression, and pain catastrophizing. Guidelines must be established to optimize intervention strategies using these devices. Additionally, while the study findings did not indicate DHIs as more beneficial for some types of pain than others, this presents a prospective avenue for further research to explore. 

Limitations 

Limitations identified in this scoping review encompassed restricting the evaluated conditions to those falling in the most common chronic pain conditions: musculoskeletal conditions (arthritis, osteoarthritis, LBP), fibromyalgia, migraines and headaches, and endometriosis. Additionally, articles were confined to those published in the English language. While there was considerable ethnic diversity in participants, socioeconomic disparities related to the accessibility of these technologies may introduce potential bias. Furthermore, the exclusion of studies involving children and adolescents, who have increasing technological proficiency and accessibility, but who are historically less commonly affected by chronic pain conditions, perpetuates a lack of knowledge regarding the potential impact of DHIs on children and adolescents living with chronic pain conditions. 

Many of the studies focused on one specific chronic pain condition and/or a lack of randomization/selection bias among patients where participants were non-blind to being in the experimental group or were selected based on having a diagnosis of a particular condition [36,43-45,47,48,55,62,64]. Furthermore, several studies identified additional constraints such as small sample sizes [36,38,39,48,49,52,55,61,62,64], gender imbalances [42,55,61,68] lack of generalizability [47,55,61,62,68], patient adherence concerns [36,39,49,50,64], and technical challenges [43,47,64]. Some articles also raised concerns about patient self-reporting, which may have impacted the accuracy of the findings without an objective assessment [39,41,42,44,60,62]. Additionally, the recent development of many assessed DHI technologies led to concerns about the lack of long-term follow-up in several studies [37-39,45,47,51,53,60,64]. 

Lastly, only a limited number of databases were utilized in the creation of this scoping review with additional resources also available that could have yielded more information to bolster arguments and examine common themes for the use of DHIs in chronic pain management. The typical limitations of scoping reviews must also be considered as this paper prioritized encompassing data that provided overarching themes of DHIs rather than focusing on the depth of individual chronic pain topics and the efficacy DHIs provide in their management. Future research is needed into individual disorders of chronic pain to establish more nuanced and precise insights into the efficacy of DHIs in their therapeutic interventions.

## Conclusions

This scoping review highlights the diverse landscape of interventions targeting pain reduction in chronic pain management with themes of pain reduction, increased accessibility and cost-effectiveness, positive mental health implications, and pain catastrophizing. Despite continued progress, ongoing research is needed to understand the underlying mechanisms of mental health implications and the potential economic benefit posed by DHIs. This is essential for advancing the understanding of effective strategies for managing chronic pain and ultimately enhancing patient outcomes. The findings underscore the importance of tailored approaches and ongoing research efforts to optimize pain management outcomes. 
